# Supplementary material for: On the Comparison of Records from Standard and Engineered Fiber Optic Cables at Etna Volcano (Italy)
Source: Sensors (Basel). 2023 Apr 4;23(7):3735. doi: 10.3390/s23073735 (PMC10099261; doi:10.3390/s23073735)
Supplement: Supplementary file 1 [file sensors-23-03735-s001.zip › sensors-2272184-supplementary.pdf]

On the comparison of records from Standard and Engineered fiber optic cables at Etna volcano (Italy)

Supplementary Material

April 1, 2023

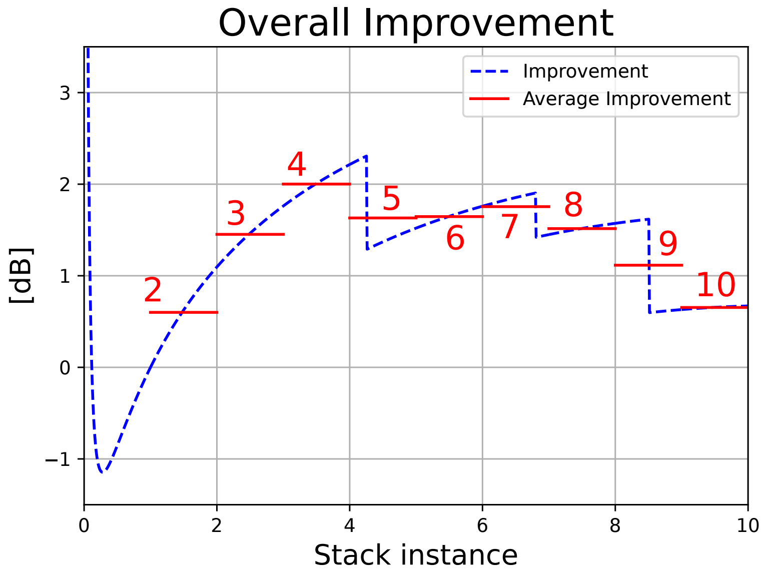

Figure 1: Overall Improvement curve in dashed blue from formula derived in Appendix. Red lines represents the values of the Overall Average Improvement for the specified fiber instance. Calculations were done for a fixed sampling frequency of 1 kHz.

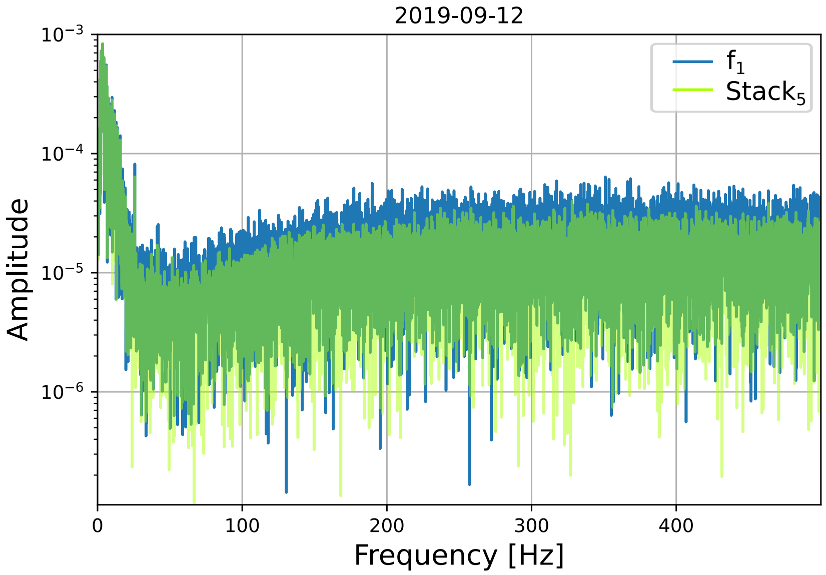

Figure 2: Spectrum of stack instance 5 of standard fiber and fiber instance 1 in green and blue curves, respectively.

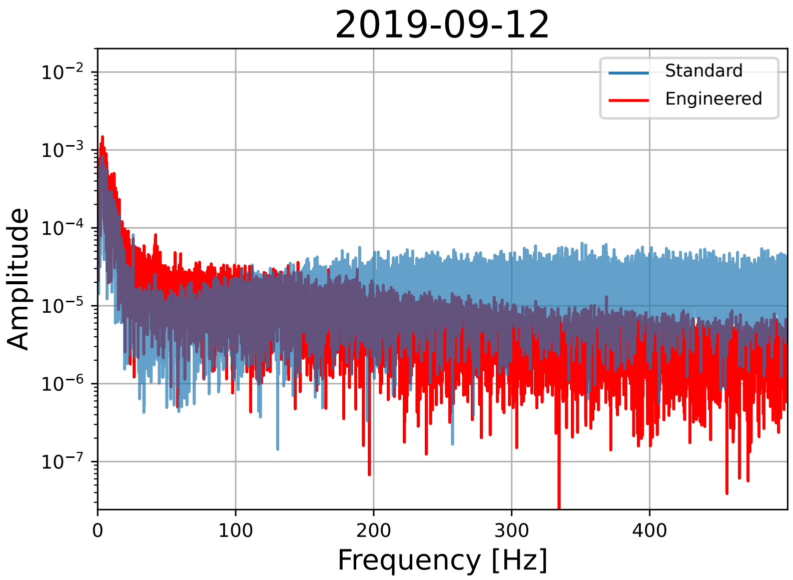

Figure 3: Spectrum of standard fiber (fiber instance 1) and engineered fiber in blue and red curves, respectively.

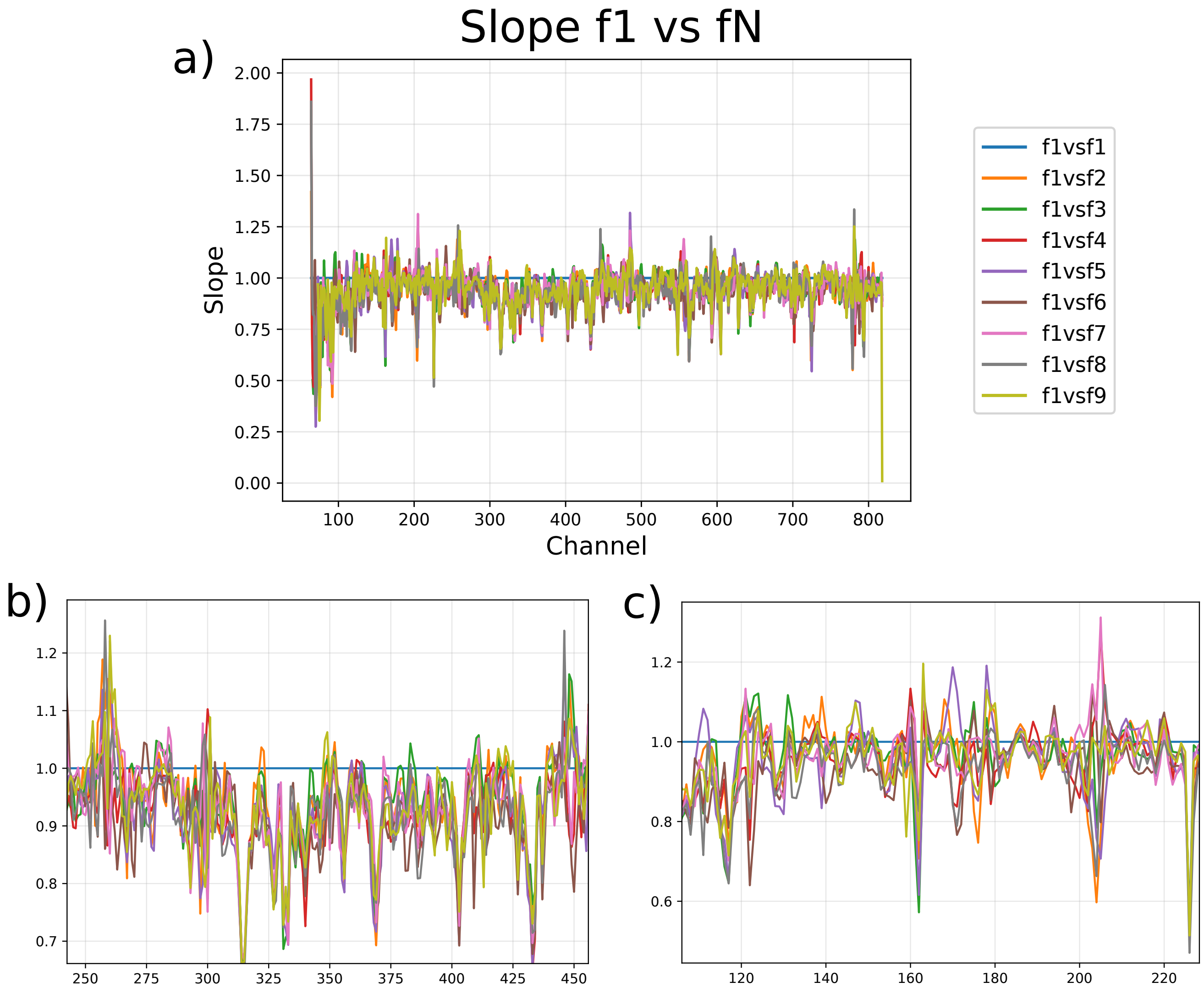

Figure 4: Slope between fiber instance 1 against the stack instances across all channels. Reduction of amplitude is shown as the overall slope is reduced below 1. **a)** is the general view across all channels, while **b)** and **c)** are zoomed sections.

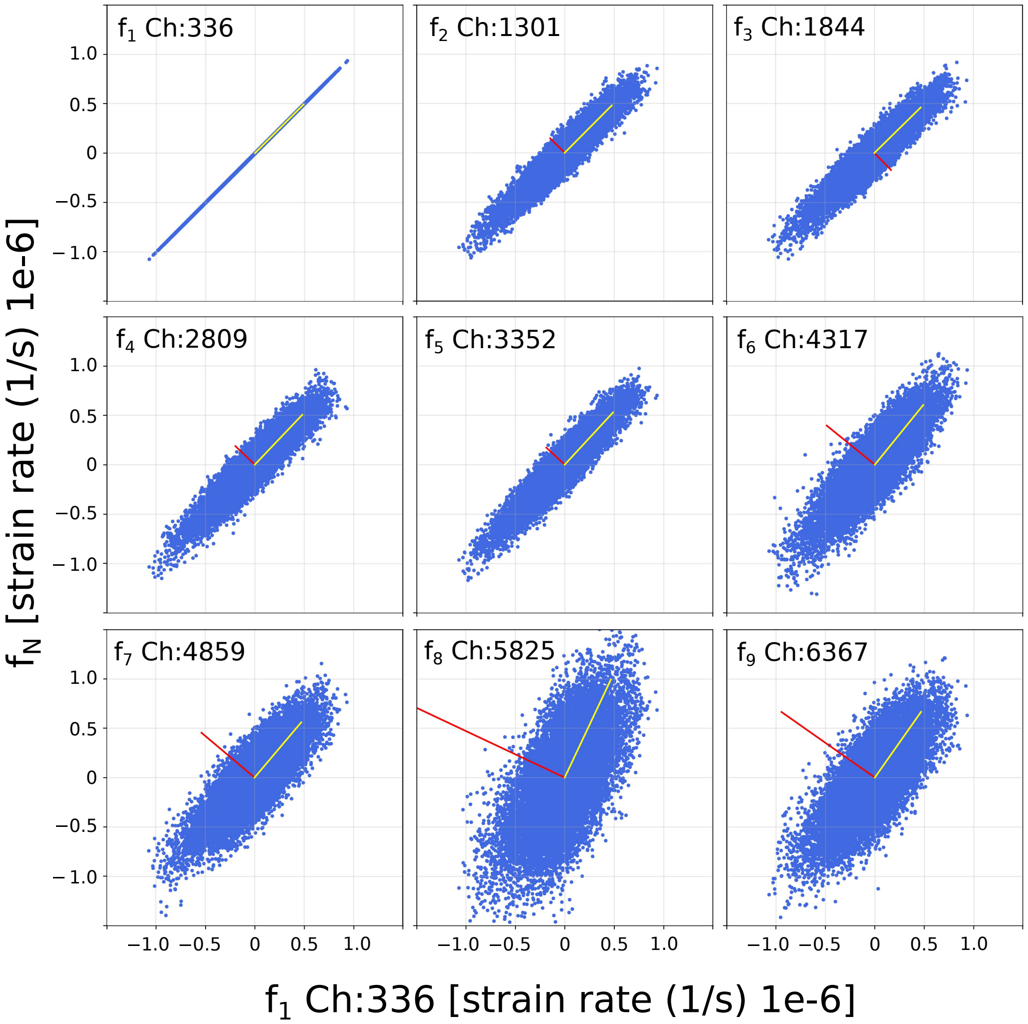

Figure 5: Strain-rate cross-plots in lowpass filter between the standard fiber instance 1 and other fiber instances amplitudes using the equivalence of channels 336 (Standard) to the other ones indicated on each fiber instance. Yellow and red lines indicates the direction of the eigenvectors $\overrightarrow{e_1}$ and $\overrightarrow{e_2}$ for the specific fiber 1 instance vs. the other instances amplitude comparison, respectively.

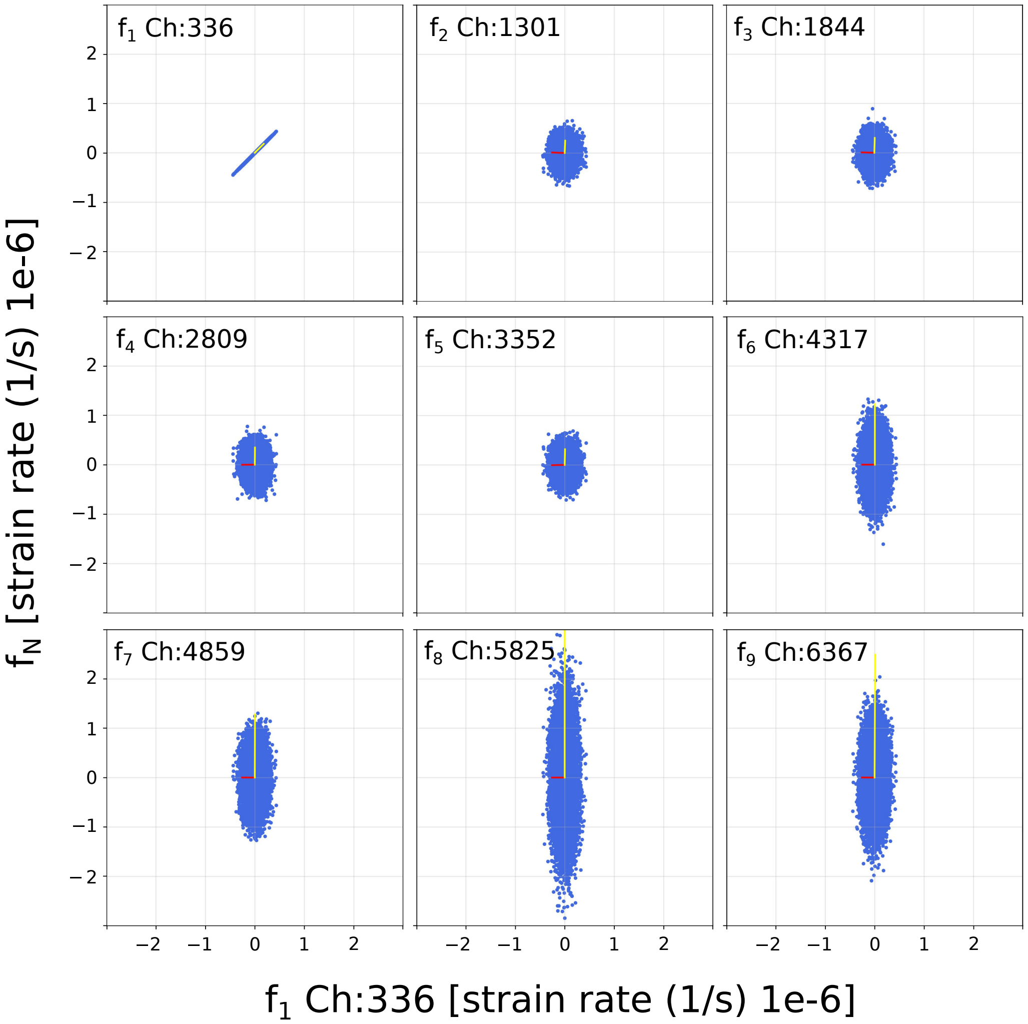

Figure 6: Strain-rate cross-plots in highpass filter between the standard fiber instance 1 and other fiber instances amplitudes using the equivalence of channels 336 (Standard) to the other ones indicated on each fiber instance. Yellow and red lines indicates the direction of the eigenvectors $\overrightarrow{e_1}$ and $\overrightarrow{e_2}$ for the specific fiber 1 instance vs. the other instances amplitude comparison, respectively.

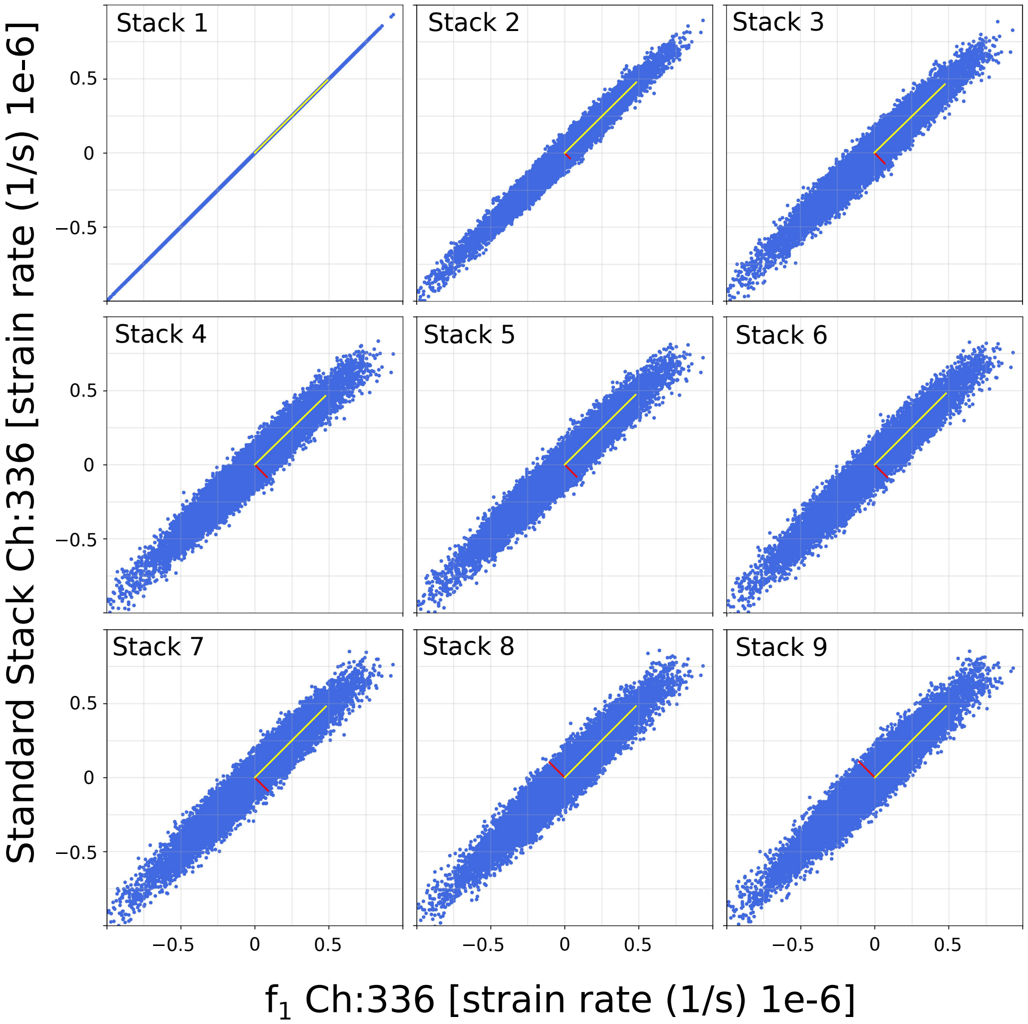

Figure 7: Strain-rate cross-plots in lowpass filter between the standard fiber instance 1 and stack instances amplitudes using channel 336. Yellow and red lines indicates the direction of the eigenvectors $\overrightarrow{e_1}$ and $\overrightarrow{e_2}$ for the specific fiber 1 instance vs. the other instances amplitude comparison, respectively.

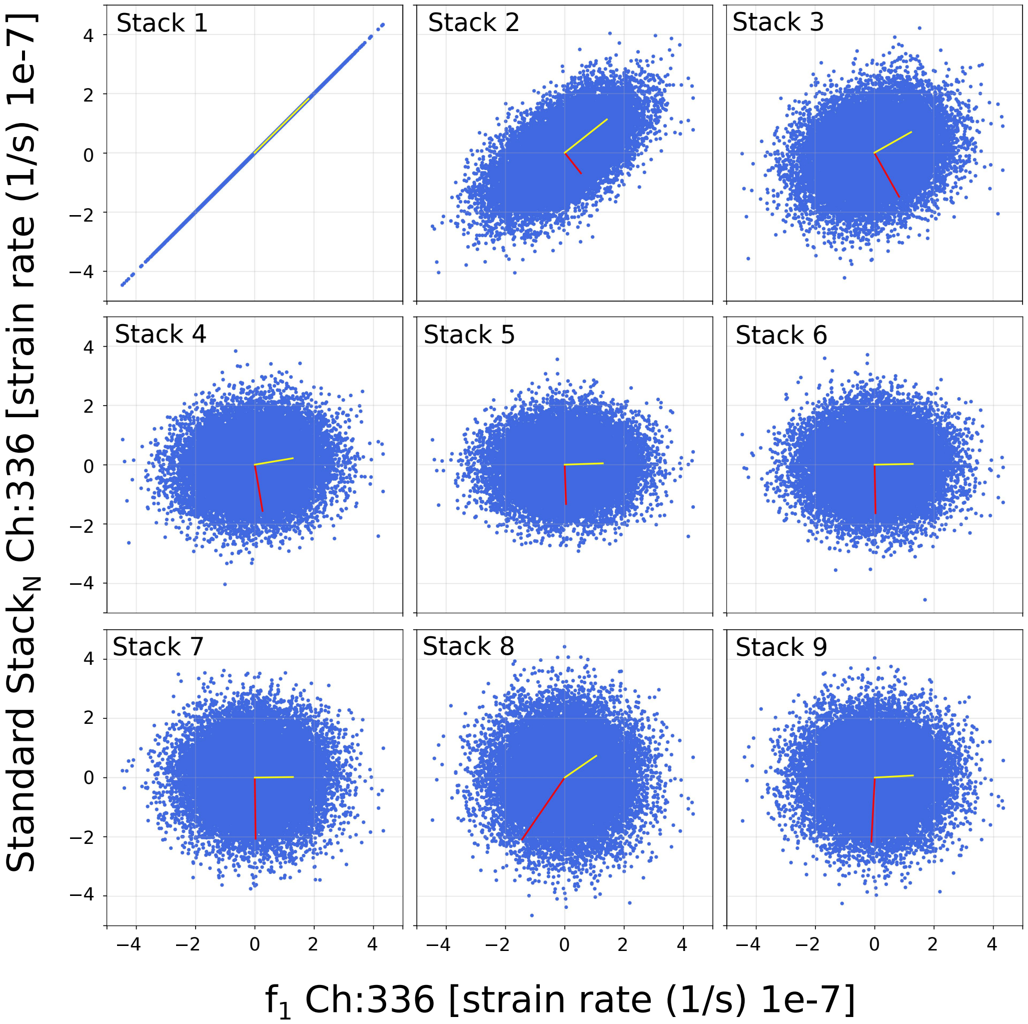

Figure 8: Strain-rate cross-plots in highpass filter between the standard fiber instance 1 and stack instances amplitudes using channel 336. Yellow and red lines indicates the direction of the eigenvectors $\overrightarrow{e_1}$ and $\overrightarrow{e_2}$ for the specific fiber 1 instance vs. the other instances amplitude comparison, respectively.

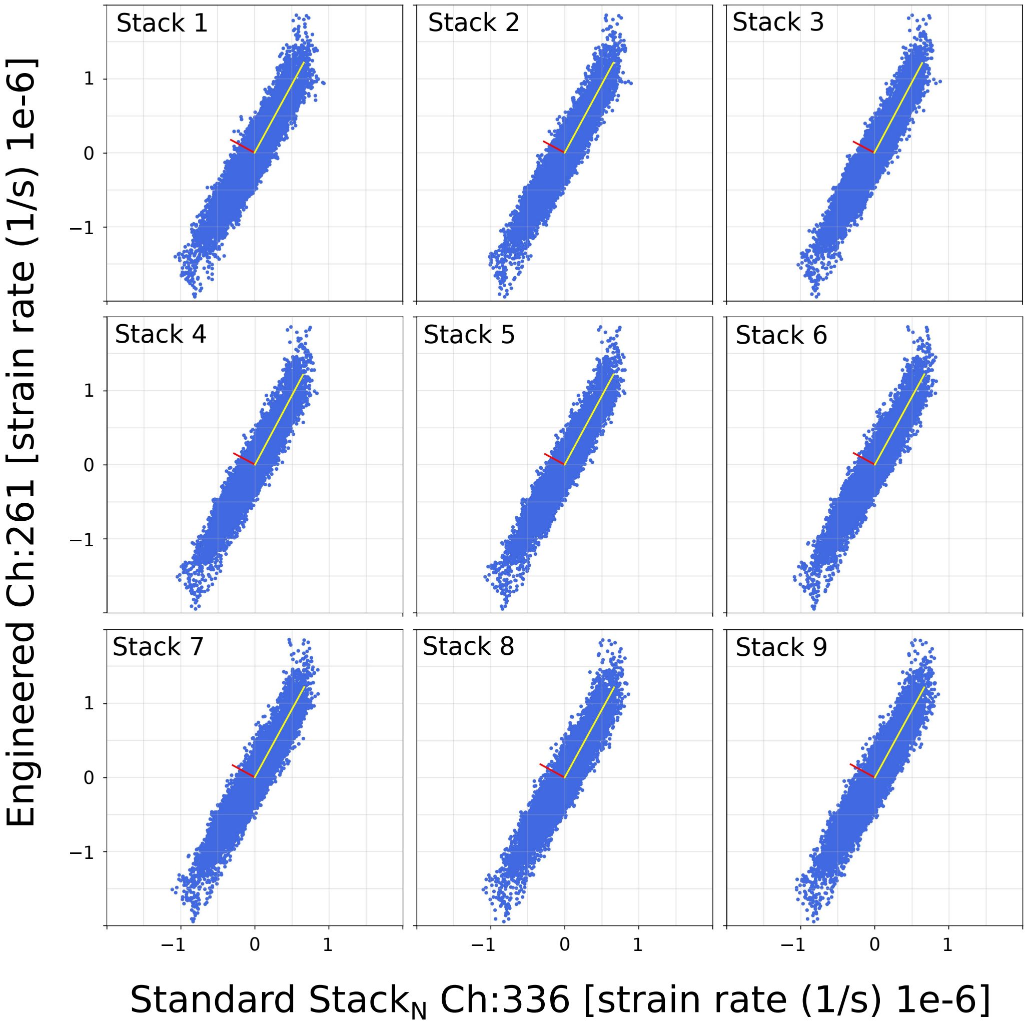

Figure 9: Strain-rate cross-plots in lowpass filter between the standard stack instances and engineered amplitudes using equivalent channels 336 and 261 for standard and engineered fiber, respectively. Yellow and red lines indicates the direction of the eigenvectors $\overrightarrow{e_1}$ and $\overrightarrow{e_2}$ for the specific fiber 1 instance vs. the other instances amplitude comparison, respectively.

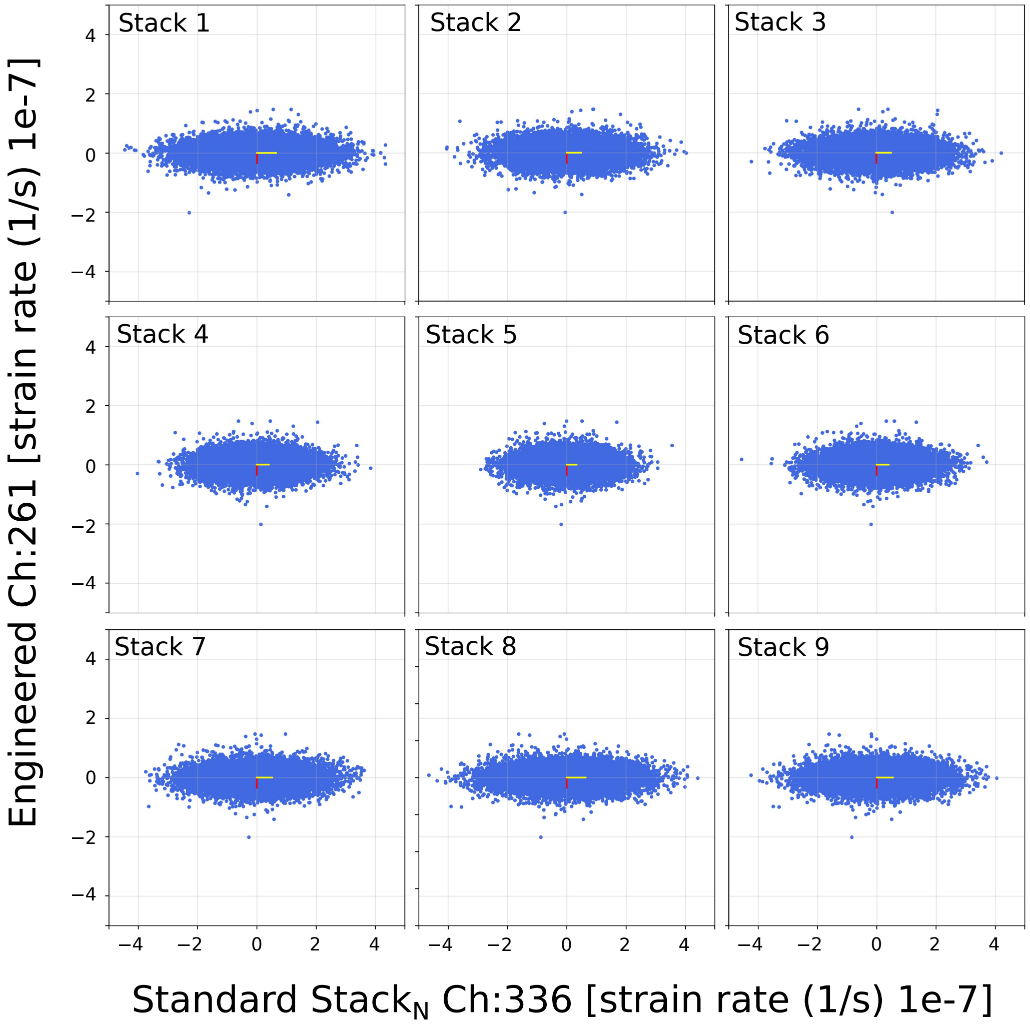

Figure 10: Strain-rate cross-plots in highpass filter between the standard stack instances and engineered amplitudes using equivalent channels 336 and 261 for standard and engineered fiber, respectively. Yellow and red lines indicates the direction of the eigenvectors $\overrightarrow{e_1}$ and $\overrightarrow{e_2}$ for the specific fiber 1 instance vs. the other instances amplitude comparison, respectively.

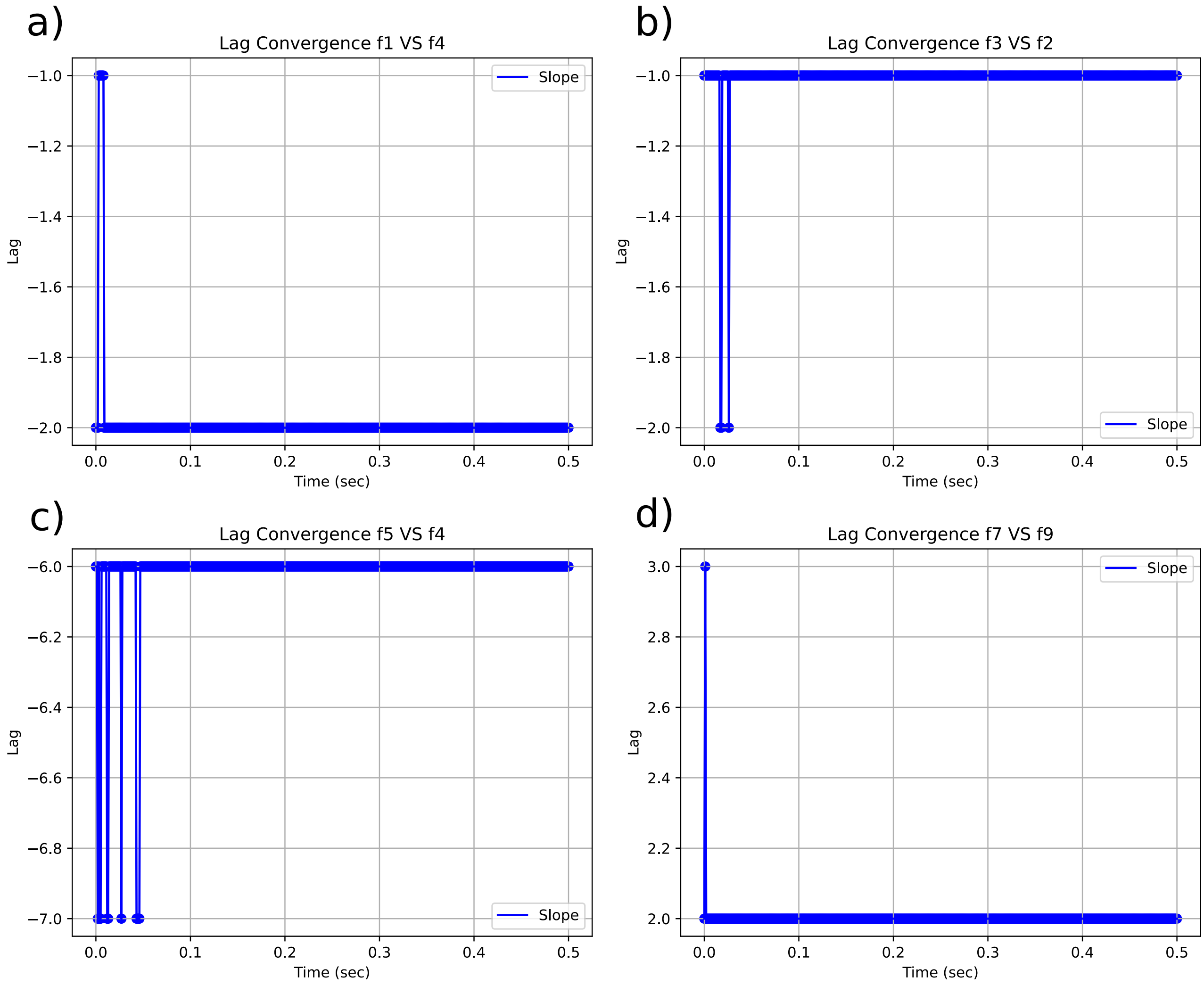

Figure 11: Spatial CC convergence over time-sample included in the Spatial CC for fiber instances pairs. Only 4 random examples are included which are: **a)** fiber instances 1 and 4, **b)** fiber instances 3 and 2, **c)** fiber instances 5 and 4, **d)** fiber instances 7 and 9.

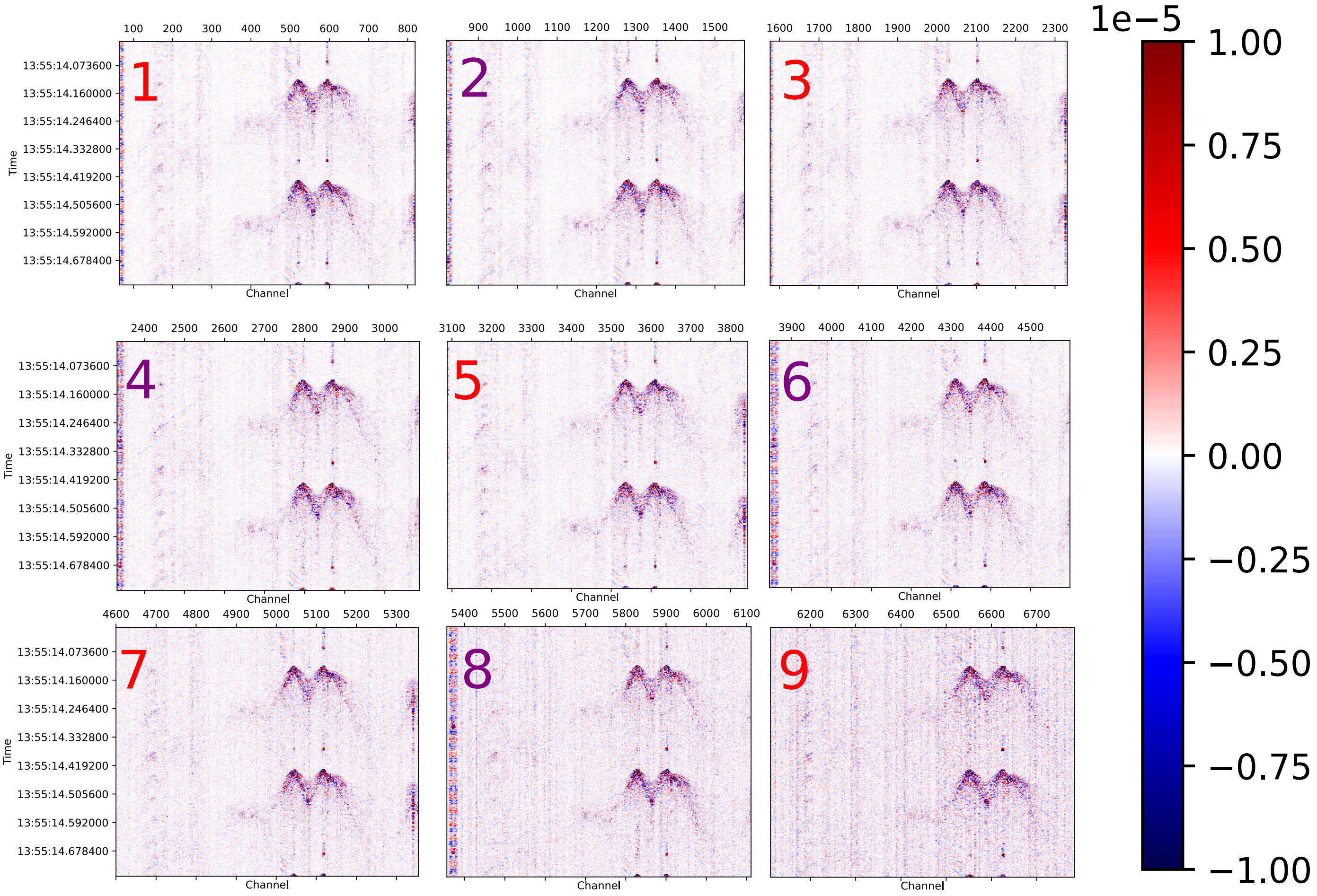

Figure 12: Windows of Standard fiber instances from Figure 3 in the main text. The purple numbers in the upper-left part of the windows indicate the windows that were reflected for the Spatial CC (and show reflected here) while the red numbers indicate windows in the original orientation (unaffected).

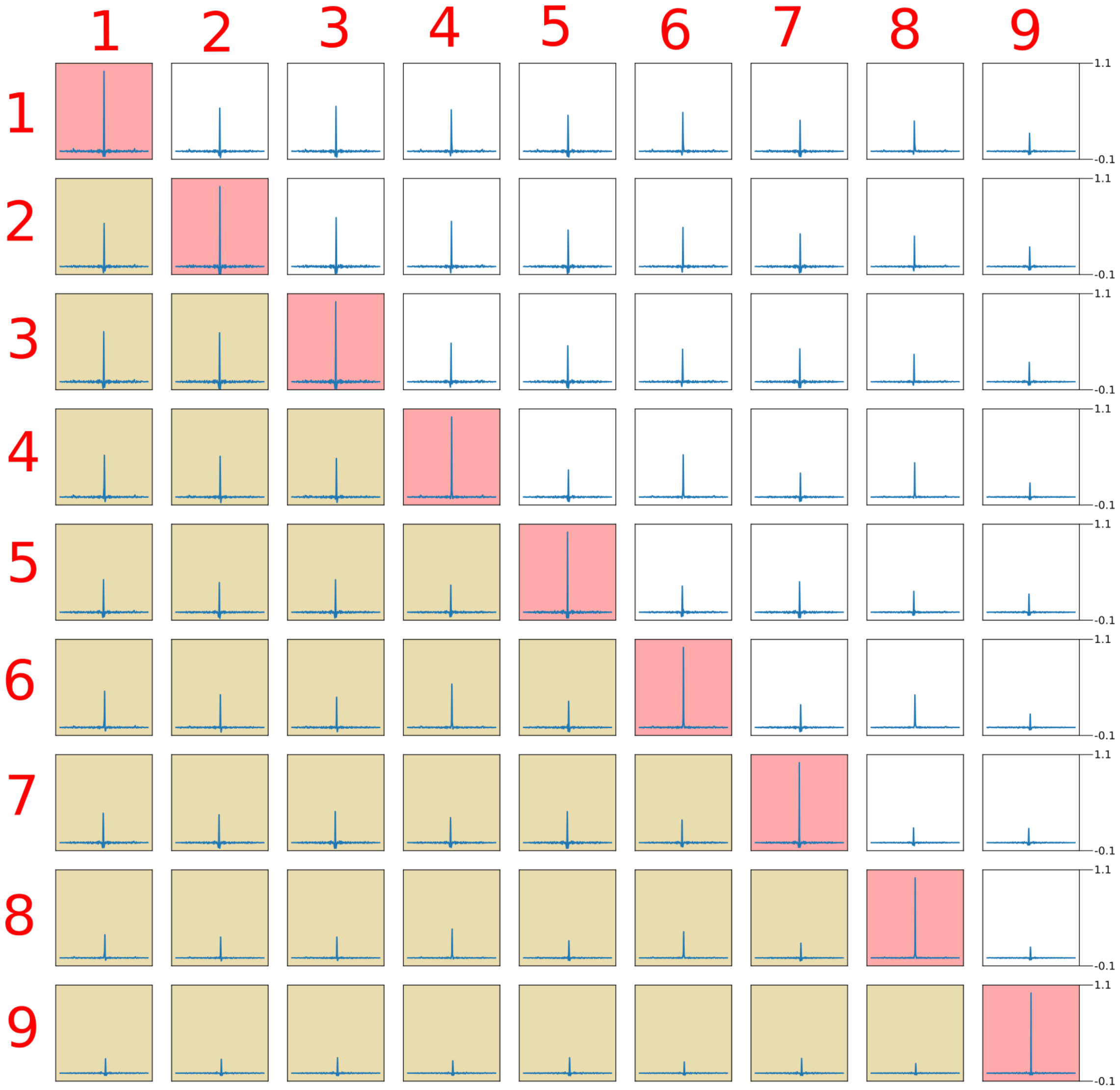

Figure 13: Visualization of Spatial CC functions achieved for fiber instance pairs. Red numbers indexes indicate the fiber instance number of the CC matrix. Plots with red background are auto-correlations (diagonal components) and light brown are the reflected CC functions of the white areas.

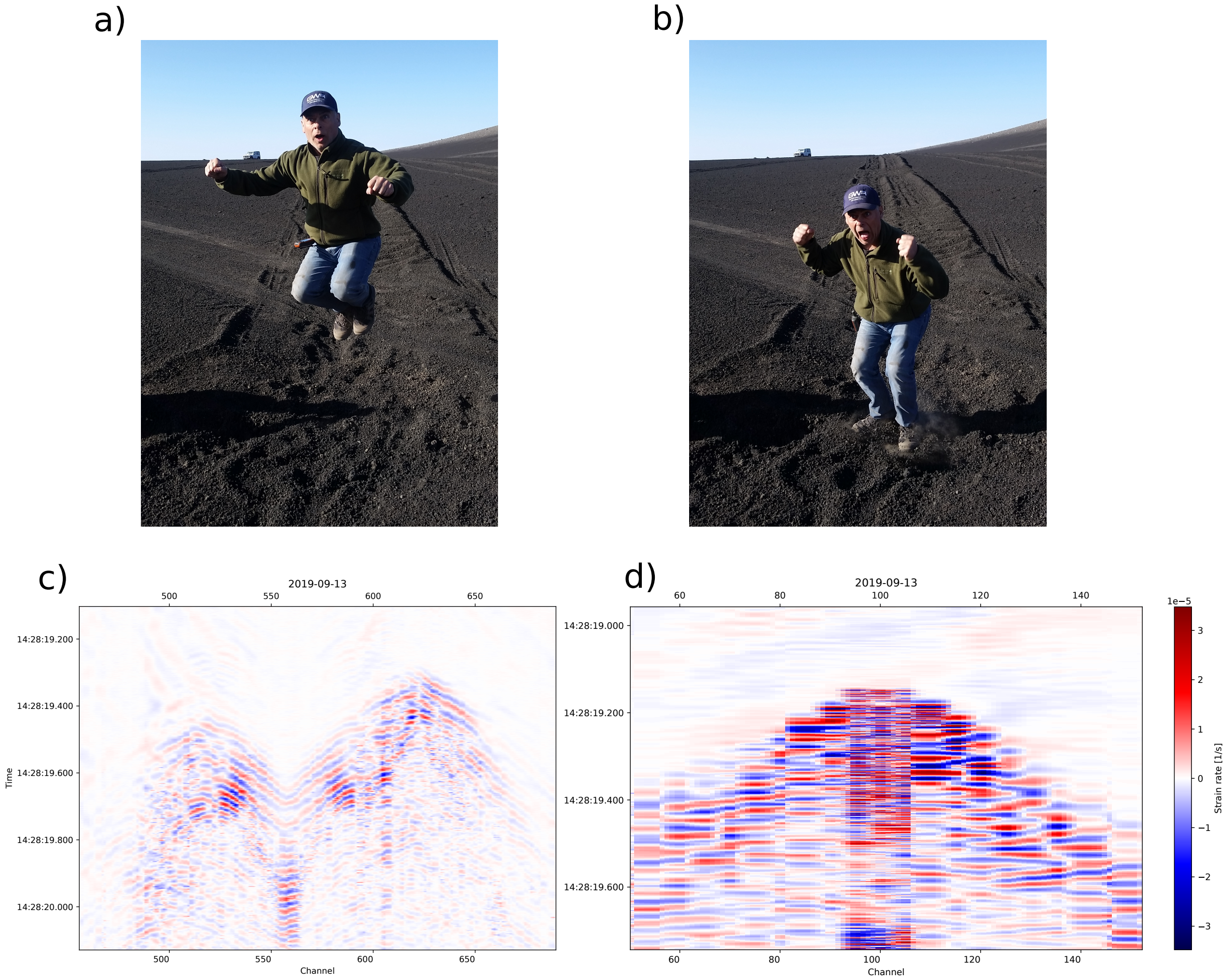

Figure 14: Example of a tap test (JJ29, see Table 2) as **a)** jumping above the ground and **b)** landing back. Subfigures **c)** and **d)** show the recorded tap in standard and engineered fibers, respectively. Tap location was near engineered fiber, but far from standard fiber. Standard cable crosses perpendicularly 2 times the direction of the engineered fiber, and so 2 records of the tap test are seen with a time delay for 2 ranges of channels.

**Variable**	**iDAS (standard fiber)**	**Carina (engineered fiber)**
Sampling Frequency[Hz]	1000.0	1000.0
Spatial Resolution[m]	2.0	1.0
Start Position[m]	167.2742	412.0
Measure Length[m]	13824	576
Stream Time[s]	inf	inf
Continuous Mode	True	True
Source Mode	Normal	Normal
Power Decrement	0.0	0.0
Start Distance (m)	-124.020952	1549.805719
Stop Distance (m)	13987.6248	2136.85334
Precise Sampling Frequency (Hz)	5000.0	16000.0
Peak Voltage[V]	2.0	2.0
Pulse 2 Delay (ns)	500.0	40.0

Pulse Width [ns]	50.0	140.0
OffsetLength	64.0	64.0
PreTrig Samples	94	64
Reference length	0	1
Saving Bandwidth (MB/s)	350.0	350.0
Reference Level 1	65535	65535
Reference Level 2	50631	52385
Reference Level 3	50215	58016
Fibre Index	1.4682	1.4682
Fibre Length Multiplier	1.0209523838714072	1.0209523838714072
Unit Calibration (nm)	116.0	116.0
Diff Amplify Factor	0	0
Attenuator 2	2.31502	2.070873
Fibre Length per Metre	1.0	1.0
Zero Offset (m)	-124.02095	1549.805719
Receiver Gain	147.00 147.00 147.00	0 0 0
Pulse Width 2 (ns)	50.0	40.0
Peak Voltage 2 (V)	0.0	0.0
Tags	NA	NA
Gauge Length	10.0	10.209523838714073
Attenuator 1	0.0	2.206218
Time Decimation	5	16
Output Decimation [bool]	2	2
P	5	11
P Coefficients	1.0 x 5	1.0 x 11
Integration Cut Off (Hz)	3.14	3.14
Normalization	True	True
Decimation Filter	True	True
Acoustic Output	Differential	Differential
Diagnostic Output	R, S, As & dP	R, S, As & dP
Window	0	0
GPS TimeStamp	2019-09-12T11:56:06.859969	2019-09-11T04:24:28.340626
Defragmented	False	False
CPU TimeStamp	2019-09-12T12:56:06.859+01:00	2019-09-11T04:25:02.156365
iDAS Version	2.4.1.102	2.5.0.88
iDAS ClockSource	AA Onboard PLL	AA Onboard PLL
ISO8601 Timestamp	2019-09-12T12:56:06.859+01:00	2019-09-11T04:24:28.340+00:00

Table 1: Metadata variables for the survey done with iDAS and Carina units. The 2 metadatas correspond to the first files generated for each survey. No change is expected since adquisition was continuous.

Tap ID	Time (UTC)	Latitutde	Longitude	lat N (UTM)	lon E (UTM)	Altitude (m.a.s.l)	Channel Standard	Channel Engineered
JJ01	10:51:21	37.766485	15.0156	4179906.675	501373.97	2799.065	134	NA
JJ02	10:53:33	37.766276	15.015213	4179883.482	501339.889	2791.252	155	NA
JJ03	10:56:32	37.766363	15.014783	4179893.128	501302.015	2788.954	174	NA
JJ04	10:59:15	37.76641	15.01433	4179898.336	501262.116	2798	194	NA
JJ05	11:01:54	37.766502	15.01378	4179908.536	501213.673	2791.259	219	497
JJ06	11:06:30	37.766645	15.012942	4179924.391	501139.864	2793.577	255	423
JJ07	11:11:43	37.76677	15.012175	4179938.251	501072.309	2795.34	290	355
JJ08	11:16:00	37.766808	15.011948	4179942.464	501052.315	2795.259	300	335
JJ09	11:19:09	37.766648	15.01185	4179924.711	501043.686	2796.014	312	316
JJ10	11:23:43	37.766133	15.011581	4179867.57	501020.001	2796.757	342	256
JJ11	13:45:38	37.76548	15.011324	4179795.119	500997.375	2798.106	380	182
JJ15	13:50:44	37.764345	15.0108	4179669.188	500951.237	2804.156	448	NA
JJ19	13:55:13	37.764803	15.012357	4179720.019	501088.367	2794.821	521	NA
J14 july	14:00:05	37.76492	15.01272	4179733	501120.34	2797	537	45
J15 july	14:01:43	37.76503	15.01311	4179745.21	501154.69	2795	560	NA
JJ20	14:05:43	37.764828	15.012165	4179722.79	501071.456	2795.611	606	NA
JJ21	14:07:49	37.765152	15.012586	4179758.742	501108.532	2794.248	631	66
JJ22	14:10:56	37.765763	15.013643	4179826.544	501201.619	2793.034	688	NA
JJ24	14:13:32	37.766213	15.014338	4179876.48	501262.824	2791.077	726	NA
JJ25	14:16:40	37.765261	15.013874	4179770.852	501221.973	2791.612	780	NA
JJ27	14:19:47	37.765102	15.013414	4179753.205	501181.46	2794.656	804	NA
JJ28	14:22:38	37.76507	15.013143	4179749.651	501157.592	2796.251	818	NA
JJ29	14:28:17	37.765256	15.012211	4179770.276	501075.502	2800.418	622	100

Table 2: GPS measurements of the tap tests carried out along the path of the Standard and Constellation cables.

Fibre Type	Fibre instance	Channel No.
Standard	1	336
	2	1301
	3	1844
	4	2809
	5	3352
	6	4317
	7	4859
	8	5825
	9	6367
Engineered	1	261

Table 3: Equivalent channels between channel 261 in engineered fibre and the other standard fibre instances.
